# Cardiovascular disease, diabetes and established risk factors among populations of sub-Saharan African descent in Europe: a literature review

**DOI:** 10.1186/1744-8603-5-7

**Published:** 2009-08-11

**Authors:** Charles Agyemang, Juliet Addo, Raj Bhopal, Ama de Graft Aikins, Karien Stronks

**Affiliations:** 1 Department of Social Medicine, Academic Medical Centre, University of Amsterdam, Amsterdam, The Netherlands; 2 Department of Epidemiology and Population Health, London School of Hygiene and Tropical Medicine, Keppel Street, London, UK; 3 Division of Community Health Sciences, Public Health Sciences Section, University of Edinburgh, Teviot Place, Edinburgh, UK; 4 Department of Social and Developmental Psychology, Faculty of Social and Political Sciences, University of Cambridge, Free School Lane, Cambridge CB2 3RQ, UK

## Abstract

**Background:**

Most European countries are ethnically and culturally diverse. Globally, cardiovascular disease (CVD) is the leading cause of death. The major risk factors for CVD have been well established. This picture holds true for all regions of the world and in different ethnic groups. However, the prevalence of CVD and related risk factors vary among ethnic groups.

**Methods:**

This article provides a review of current understanding of the epidemiology of vascular disease, principally coronary heart disease (CHD), stroke and related risk factors among populations of Sub-Sahara African descent (henceforth, African descent) in comparison with the European populations in Europe.

**Results:**

Compared with European populations, populations of African descent have an increased risk of stroke, whereas CHD is less common. They also have higher rates of hypertension and diabetes than European populations. Obesity is highly prevalent, but smoking rate is lower among African descent women. Older people of African descent have more favourable lipid profile and dietary habits than their European counterparts. Alcohol consumption is less common among populations of African descent. The rate of physical activity differs between European countries. Dutch African-Suriname men and women are less physically active than the White-Dutch whereas British African women are more physically active than women in the general population. Literature on psychosocial stress shows inconsistent results.

**Conclusion:**

Hypertension and diabetes are highly prevalent among African populations, which may explain their high rate of stroke in Europe. The relatively low rate of CHD may be explained by the low rates of other risk factors including a more favourable lipid profile and the low prevalence of smoking. The risk factors are changing, and on the whole, getting worse especially among African women. Cohort studies and clinical trials are therefore needed among these groups to determine the relative contribution of vascular risk factors, and to help guide the prevention efforts. There is a clear need for intervention studies among these populations in Europe.

## Introduction

Globally, cardiovascular disease (CVD) is the leading cause of death [1]. This is particularly so in Europe, where CVD has continued to maintain its lead for several decades [1], and this is reflected in Europe's multi-ethnic populations [2]. The experiences of CVD mortality, morbidity and risk factors vary hugely among ethnic groups [2-6]. This is creating challenges for public health, epidemiology and clinical care.

Populations of sub-Saharan African descent (henceforth African descent) are at an increased risk of developing stroke compared with European descent populations (henceforth White) [2-4]. The patterning of these health inequalities is complex. There have been different suggestions on the possible causes of these inequalities, with some emphasising the genetic underpinning of such inequalities [7] and others arguing that ethnic differences in health are mainly determined by socio-economic inequalities [8-11]. Understanding the reasons behind the excess risks is crucial for addressing ethnic inequalities in health. Data by indicators of ethnic group are needed to establish the extent of health inequalities and inequity in health service provision.

This article provides a review of current understanding of the epidemiology of vascular disease, (principally coronary heart disease (CHD) and stroke), related risk factors (i.e. hypertension, diabetes, abnormal lipids, smoking and alcohol intake, obesity, dietary patterns, physical inactivity and psychosocial stress), possible causes and management, and critical gaps of knowledge among populations of Africa populations in Europe. We have chosen the risk factors found to be most important by the Inter-Heart study, which are widely recognised as the major risk factors for CHD. Collectively, these risk factors accounted for 90% of the population-attributable risk (PAR) in men and 94% in women [12]. In addition, the paper also summarises the putative emerging CVD risk factors, access and quality of care and provides recommendations for future work among these populations in Europe. The key questions to be addressed are: what is the burden of CVD and its related risk factors among populations of African descent in Europe? What are the possible reasons for the increased burden? And what are the differences in the management of risk factors for CVD between populations of African descent and their European White counterparts?

## Methods

Data from individual studies and systematic review articles known to the authors were examined. Electronic databases (MEDLINE, EMBASE and Google Scholar) searchers were also performed using combinations of the key terms 'Africans', 'African Caribbean', 'West Africans', 'Black' and 'ethnic minority groups', and were combined with cardiovascular diseases and related various risk factors. Reference lists were reviewed to identify additional relevant data sources. Key references were examined by first and second authors. Articles used in this review consist of scholarly papers published between 1960 until February 2009.

### Note on ethnicity

There is no consensus on appropriate terms for the scientific study of health by ethnicity, and published guidelines are yet to be widely adopted. We have followed general conventions used in Europe and, whenever appropriate, the terminologies used in the original documents were referred to [12]. The term 'ethnic minority group' refers to minority non-European, non-White populations [12]. Ethnicity refers to the group individuals belong to as a result of their roots, which include language, religion, diet, and ancestry [12]. Different terms are used to refer to populations of African descent living in different European countries [13]. African Caribbean refers to people, and their offspring, with African ancestral origin but who migrated to the UK via the Caribbean islands. Sub-Saharan ('black') African refers to people, and their offspring, with African ancestral origin who migrated via sub-Saharan Africa. African Surinamese is used to refer to people with African ancestral origins and their offspring who migrated to the Netherlands via Suriname.

#### Populations of African descent in Europe

The migration of populations of African descent to Europe has a long history, and the reasons of migration and the subsequent relationship between the African migrants and the European populations have been determined largely by the order of the time. Britain, for example, has a long history of contact with Africa [14]. The presence of populations of African descent in Britain has been reported since 200 AD [15]. Several hundred years afterwards the influence of the Atlantic slave trade – one of the darkest episodes in human history- began to be felt, with the first group of West Africans being brought to the UK in 1555 [14]. By the last third of the 18th century, there were an estimated 10,000 Africans in Britain [16], concentrated mostly in cities such as London.

The migration of the populations of African descent to Europe in the mid-20th century was mainly due to the need to rebuild Europe following World War II. The demands of an expanding economy and the development of the welfare state required labour on a scale that could not be provided locally. The economic downturn and the political instability in the last few decades in many African and Caribbean regions also contributed to this flow of migration from Africa and the Caribbean to Europe.

Colonial links played a major role in determining the European destination where the Africans migrated. People from the Commonwealth nations of Africa such as Nigeria and Ghana migrated to Britain whereas those from Francophone countries such as Ivory Coast and Senegal migrated to France. Similar patterns were also observed among the Caribbean groups such as Jamaicans moving to the UK and Surinamese moving to the Netherlands [17]. These patterns of migration might be partly due to the influence of colonial heritage such as language familiarity and similar educational systems.

Estimating future population size of populations of African descent is complicated and has to take into consideration not only fertility, mortality and net migration, but also ethnic identity [2]. What is obvious is that many of these populations are ageing and the burden of CVD will increase. This has major implications for health and social care.

### Epidemiology of cardiovascular disease in populations of African descent in Europe

Except for the UK, and a few reports in the Netherlands, information on CVD among populations of African descent is limited in Europe. Hence, this outline will be largely based on data from the UK and the Netherlands. The summarised results are given in Table 1. These data suggest that these populations have a high incidence of stroke [2-4], whereas CHD is less common [2-4]. These findings are consistent with the reports in the USA [16]. Despite the higher rate of stroke, survival after stroke has been shown to differ between different European countries. One UK study, for example, found better survival rates among populations of African descent than in the White group [18]. By contrast, a recent Dutch report found stroke survival rates in both short- and long-term to be poorer in all ethnic minority groups than in the White-Dutch population [19].

**Table 1:** Comparison of disease outcomes and risk factor levels among populations of African descent in the UK and the Netherlands

	**Men**			**Women**		
	African Caribbeans	Sub-Saharan Africans	African Surinamese	African Caribbeans	Sub-Saharan Africans	African Surinamese

**Disease outcomes**						

Stroke [2-5]	+	+	+	+	+	+

Coronary heart disease [2-5]	-	-	-	-	-	-

Type II diabetes [26,27,34,71]	+	+	+	+	+	+

						

**Chemical measurement risk factors**						

High total cholesterol* [26,89,71]	-	-	-	-	-	-

Low HDL cholesterol [26,89,71]	-	-	-	-	=	-

ApoA-1 [89]	=			=		

ApoB [89]	-			-		

						

**Physical measurement risk factors**						

Hypertension [23,24]	+	+	+	+	+	+

Obesity (BMI > 30 kg/m^2^) [23,24,94,97]	=	-	=	+	+	+

Abdominal obesity [23,24,94]	-	-	-	+	+	+

						

**Self-reported risk factors**						

Current smoking [93,94]	=	-	+	=	-	-

Alcohol consumption [93,94]	-	-	-	-	-	-

Physical inactivity (non-adherence to recommendations) [66,93]	=	+	+	-	=	+

Consumption of < 5 portions of fruit and vegetables* [93,110]	-	-	- **	-	-	- **

Psychosocial stress [26,122-125]	inconsistent	inconsistent		inconsistent	inconsistent	

- Lower risk than White European population

+ higher risk than White European population

= comparable risk to White European population

* applied only to the non-UK born

** the Dutch group were based on overall diet quality

The fact that studies have found a lower rate of CHD in populations of African descent than in their European counterparts [2,3] does not imply that CHD is uncommon among these populations. Heart disease still remains one of the single most important causes of death among these populations in Europe. In fact, recent data from the UK indicate that the CHD advantage is diminishing rapidly. Recent analyses by Harding and colleagues [6], spanning from 1979 to 2003, show very worrying trends. For the first time Jamaican born women had a higher directly age-standardised CHD death rate than those born in England and Wales. In 1979–83, the age-standardised rate of CHD was lower in Jamaican born women than those born in England and Wales (Rate ratio = 0.63, 95% CI: 0.52, 0.77). In 1999–2003, they were more likely than those born in England and Wales to have CHD (Rate ratio = 1.23, 95% CI: 1.06, 1.42). The gap between Jamaican born men and those born in England and Wales is also closing rapidly. The age-standardised rate ratio of CHD in Jamaican born men in 1979–83 was 0.45 (95% CI: 0.40, 0.50). In 1999–2003, the rate had increased to 0.81 (95% CI: 0.73, 0.90). The convergence of CHD rates among the UK African populations is reminiscent of what happened in the USA where African Americans now have a higher rate than the White Americans, reversing the previous pattern [20]. These changing trends may be due to the fact that the CHD rate has declined more rapidly in White populations than in ethnic minority populations.

### Established vascular risk factors

The causes of the excess stroke morbidity and mortality, and the lower CHD burden among populations of African descent are incompletely understood. The available evidence indicates that the excess CVD morbidity and mortality may due to several factors including the higher prevalence of CVD risk factors such as hypertension and diabetes [8,21-29].

The major risk factors for CVD have been well established. These include hypertension, diabetes, abnormal lipids, smoking, obesity, low consumption of fruits and vegetables, alcohol intake, physical inactivity and psychosocial stress. This picture holds true for men and women, in all age groups, all regions of the world and in all ethnic groups [11,30]. The majority of patients who develop CVD have at least one of these risk factors. In the INTERHEART study [11], these nine risk factors provided a PAR of 97.4% for myocardial infarction for the participants of African descent. The INTERHEART Africa study also showed that five modifiable risk factors (hypertension, diabetes, abdominal obesity, elevated ApoB/ApoA-1 ratio and current/former tobacco smoking) provided PAR of 89.2% for a first-time myocardial infarction [31].

#### Hypertension

##### Burden

Hypertension is highly prevalent among populations of African descent in Europe [24,25] and North America [26,32], and deserves a special detailed outline in these populations. The increased prevalence of hypertension among these populations in Europe appears to be a major contributor to the observed elevated stroke risk [2,33]. In the UK, for example, there is a consensus that the prevalence of hypertension is three to four times higher in the population of African descent than in White people [24,33-35]. This holds for both men and women. A higher prevalence of hypertension has also been reported among populations of African descent in other European countries such as the Netherlands [25]. The recent SUNSET study found that African-Surinamese men were over two times and African-Surinamese women were nearly four times more likely than their White-Dutch counterparts to have hypertension [25]. These observations fit with the higher rates of stroke among these populations in Europe [2-5]. African Americans also show an increase in the prevalence of hypertension compared to their White American counterparts in the USA [32,36]. In Africa itself, hypertension is rapidly becoming a major public health burden [37] particularly in urban centres [38,39]. The emerging data (for example 2004) show hypertension prevalence ranging from 16.5% in urban Eritrea to 33.4% in urban Ghana [38,39]. The increasing prevalence of hypertension reflects well on the increasing CVD mortality in Africa [40,41].

In addition to a high resting blood pressure (BP), nocturnal BP fall (i.e. daytime BP minus night-time BP) has been shown to be lower in populations of African descent in Europe than their White counterparts [42]. A diminished nocturnal decline in BP has independently been associated with increased stroke [43,44], left ventricular hypertrophy [45,46] and progression of renal damage [47]. All these conditions are highly prevalent in populations of African descent in Europe [2,48-50].

##### Causation

The reasons for the higher prevalence of hypertension among populations of African descent in Europe and North America have been well debated. To date, there are still no clear answers as to why hypertension is more common among these groups than among their European counterparts. Several explanations and speculations have been proposed including genetic factors [51,52]. Low renin levels found among African-Americans have been hypothesised to be the result of a genetic 'maladaptation' which benefited their earlier African-American ancestors to survive the ordeal of a transatlantic voyage under slavery, but later turned out to be harmful to survival due to the resultant avid salt retention [53]. Despite rigorous criticisms and unreliable data sources, this hypothesis has sustained some considerable degree of popular and scientific acceptance [54]. The issue of the link between skin colour and hypertension is even more complex. The positive relationship between dark skin and BP in some America studies has led some to suggest that the link is genetic [55]. In contrast, others have argued that it is a manifestation of the stress and social pressure of having a dark skin that causes the high BP [56].

The BP differences between the African and European descent populations may, in part, relate to environmental factors that may impact health, such as the residing countries' national context in terms of opportunities in life, psychosocial and lifestyle factors that may underline these differences. Clearly, one cannot underestimate the importance of genetics on health inequalities between populations. However, the importance of social structures, the communities where people live, and social factors cannot be underestimated [57,58]. The use of genetic mechanisms to explain familial aggregation of hypertension is a very good example. It is highly possible that the familial aggregation of hypertension might merely reflect environmental exposures shared within families, which, in turn, might increase the risk of developing hypertension rather than genes per se. In Cuba where ethnic barriers are said to be small, the ethnic differences in BP and management were shown to be small [59].

Another difficulty in explaining the BP differences between the African and European descent populations may relate to a general lack of recognition about the remarkable heterogeneity within the African descent groups in Europe and North America [13]. The disadvantages of the populations of African descent are not fixed across countries, generations, or across different African identities [13,23]. Recent emerging data are beginning to shed more light on the huge differences within the populations of African descent [22,23,60-62]. Cooper and colleague [22], for example, examined patterns of BP distribution in different ethnic groups across three continents and found a wide variation in the prevalence of hypertension both within and between the populations of African and European descent. The rates among African populations were not unusually high when compared internationally. They therefore suggest that the impact of environmental factors among African and European populations may have been under-appreciated. The recent UK studies have also revealed important heterogeneity in BP patterns between children and adults among different ethnic groups [24,60,61]. In children of African descent, BP levels were either lower [60] or similar [61] to their White counterparts in the UK. In adults, BP levels were higher in people of African descent than in White people [24]. The emerging findings clearly favour environmental or an interaction between genetics and environmental factors rather than only genetic factors per se, for it is hard to imagine genetic factors where the effect is delayed to later adult life [61]. The findings also suggest that inferences from cross-sectional studies done in certain geographic areas with different socio-cultural, economic, political and historical context cannot be extrapolated as logical benchmarks for other areas. As a result, some commentators have challenged researchers to re-examine the evidence [63].

##### Management

One of the main central focuses of the primary prevention of CVD has been increasing awareness, treatment and control of patients with hypertension. This has had a positive impact on CVD prevention in many countries [26,64,65], especially in the USA where the effort had been greatest. [26,65] Detection and treatment of hypertension appears to be similar or higher among populations of African descent than their White counterparts in Europe [34,66]. However, BP control tends to be poorer among African populations than their White counterparts [34,66]. In the SUNSET study, African-Surinamese men (odds ratio = 0.3, 95% CI: 0.1, 0.7) and women (odds ratio = 0.5, 95% CI: 0.3, 0.9) were less likely than their White-Dutch counterparts to get their hypertension adequately controlled [66]. The reasons for the low BP control among the populations of African descent are unclear, but an inadequate drug therapy owing to individual sensitivity to different drugs, non compliance with therapy, clinicians' perceptions, organisational pitfalls and cultural factors may contribute to the poor BP control found among these populations [66-68].

With concerted efforts, better BP control could be achieved for the populations of African descent in Europe [69,70]. In some trials, when medications and provider services were provided free of charge as in the Hypertension Detection and Follow-up Program, African-American men treated with the intensive "Stepped-Care Approach" actually benefited more than White Americans [70]. In a recent Jackson Heart Study report, BP control rate in African Americans was 66.4% [69]. This was comparable to the control rate of White Americans in NHANES study [26]. The control rate in African Americans in the Jackson Heart study [67] far exceeds rates reported among both African and European descent populations in many European countries [34,64,65].

#### Type 2 diabetes mellitus

##### Burden

Populations of African descent have an increased risk of type II diabetes compared with their European descent counterparts in Europe [27,28,34,71]. In the Health Survey for England (HSE) 1999, the age-standardised risk ratio for diabetes was 2.5 for African Caribbean men and 4.2 for African Caribbean women [27]. Recent Diabetes UK estimates for prevalence rates indicate that 17% of the African Caribbean community in the UK has type II diabetes compared with 3% of the UK general population [28]. The Dutch data also show a higher prevalence of type II diabetes in African Surinamese than in the White-Dutch group [71]. In a recent Dutch report [71], the age-standardised prevalence of type II diabetes in African Surinamese was 14.2% compared with 5.5% in White-Dutch individuals. The difference was more pronounced in the older age group. In the age-group 35 to 44 years, the sex-adjusted odds ratio was 1.9 (95% CI: 0.8, 4.6) for African Surinamese as compared to the White-Dutch group. In the age group 45 to 60 years, the sex-adjusted odds ratio was 2.7 (95% CI: 1.6, 4.6) for African Surinamese. Higher prevalence of diabetes had also been reported among African Americans than among White American in the USA [72]. Evidence also suggests that the prevalence of diabetes is rising rapidly in Africa with prevalence rates ranging from 0.7% in Cameroon to 8.8% in South Africa among rural dwellers, and from 1.7% in Cameroon to 10.4 in Sudan among urban dwellers [73]. In a recent review among Ghanaians and Nigerians, diabetes seemed rare in urban Ghana in 1963 (0.2%) and in urban Nigeria in 1985 (1.65%). However, in 1998, the prevalence of diabetes among Ghanaians was 6.3% and 6.8% among Nigerians [74].

##### Causation

Several factors have been linked to the increased prevalence of type II among populations of African descent such as increasing obesity, insulin resistance, physical inactivity and unhealthy diet [75-77]. Obesity is an important contributing factor to increased insulin concentrations and decreased insulin sensitivity [78]. Evidence from prospective studies indicates that the risk of type II diabetes increases progressively from a BMI of > 20 kg/m^2 ^[79-81]. Obesity is highly prevalent among populations of African descent in Europe, particularly among women (see section 4.4). In Lipton and colleagues' studies, the excess risk of type II diabetes in African Americans relative to White Americans increased with increasing level of obesity, particularly for African Americans women [82]. Insulin resistance was also higher in African Caribbeans than in Whites [77]. Insulin resistance was shown to increase the risk of both type II diabetes [83] and CVD [84].

##### Management

Several trials have shown that reducing the progression to type II diabetes in high risk groups is possible and practical irrespective of ethnicity. The American diabetes prevention programme [85], the Da Qing study in China [86], and the Finnish diabetes prevention study [87], all showed that the prevention of diabetes is feasible through diet and exercise interventions in people with impaired glucose tolerance. In the UK Prospective Diabetes Study (UKPDS), after adjusting for age, sex, baseline characteristics, treatment allocation, and change in weight, there were no consistent ethnic differences in mean change in fasting plasma glucose or HbA1c during the nine year follow up. African Caribbean patients maintained the most favourable lipid profiles, but hypertension developed in more African Caribbean patients than in White patients [88]. These data demonstrate that in a clinical trial, African Caribbeans did just as well or better than White people, even if their burden of the disease is high. A UK cohort study [89] showed lower prevalence of microvascular and macrovascular complications in African Caribbeans compared to White people over 20 years of follow up. African Caribbeans with type II diabetes maintained a low risk of heart disease [89].

The evidence to date, however, suggests that diabetes control is poorer in some populations of African descent than their European counterparts in the UK. In the Wandsworth Prospective Diabetes Study in the UK, the proportion of patients reaching treatment targets for HbA1c was significantly lower in the African Caribbeans than in White patients [90]. This may, in part, relate to poor knowledge about the disease, complications, and the importance of self management, as a result of poor communication and provision of culturally inappropriate information [91]. A study at the Manchester diabetes centre showed deficiencies in the care of African Caribbean patients compared with White patients [92].

#### Lipids

##### Burden

Populations of African descent, while having a high risk of hypertension and diabetes, have a more favourable lipid profile. In a UK population-based study that compared the lipid profile of ethnic minority groups and the general population, African Caribbeans were demonstrated to have lower levels of total cholesterol and triglycerides and higher levels of HDL cholesterol [27]. The Whitehall study of London-based civil servants reported significantly lower cholesterol, Apo B and triglyceride levels in African Caribbeans compared to White people, after adjusting for potentially confounding factors [93]. African Caribbeans had higher HDL cholesterol levels in every grade of employment than their White counterparts. The Dutch data also indicate that the African populations in the Netherlands have a more favourable lipid profile than their White-Dutch counterparts [71]. Notwithstanding this, recent evidence suggests that the favourable lipid profile among African populations in Europe is not uniform across all the populations of African descent. The analyses of the UK-born African Caribbean group indicate that lipid measures did not differ from that of the general population, except for higher HDL levels in UK-born African Caribbean men [94]. The better lipid profile among populations of African descent suggests that this factor may not contribute to their increased risk of CVD. This might change if the lipid profile deteriorates over time.

##### Causation

The reasons for the favourable lipid profile among populations of African descent are unclear. Some have suggested that these differences in lipoprotein levels are associated with genetic variations in hepatic lipase, such as populations of African descent having a higher prevalence of less active hepatic lipase phenotype and a lower prevalence of central obesity than European populations for the same degree of BMI [95]. The lack of differences between the UK-born African Caribbean group and the UK general population suggests that environmental factors may be at work [94]. Older African Caribbean group in the UK eat more traditional diets associated with a protective effect for CHD, with high fresh fruit and vegetable content, but younger UK-born African Caribbeans have greater energy intake from fat [96]. In addition, central obesity is not uniformly low among all populations of African descent [97,98]. In the Dutch SUNSET study, African Surinamese women were more centrally obese than their White-Dutch counterparts [98].

##### Management

Evidence from primary and secondary prevention trials has established that lowering LDL-cholesterol levels will lead to a substantial reduction in the risk of CVD events. Despite this, there is a paucity of data on ethnic differences in management of dyslipidemia in Europe [33]. In one USA study, African Americans were less likely than White Americans to be treated and controlled for dyslipidemia [99]. Ethnic inequalities were abolished after differences in healthcare access had been adjusted for [99]. The loss of the comparatively favourable lipid profile among the UK-born African Caribbeans clearly indicates the need to monitor lipid profiles among these populations in Europe [96].

#### Overweight and obesity

##### Burden

Overweight and obesity are highly prevalent among populations of African descent in Europe, especially among women [24,25,97,98,100]. In the HSE 2004 [97], the prevalence of overweight and obesity were 32.4% and 32.1% in the African Caribbean women and 31.3% and 38.5% in the Sub-Saharan African women as compared with 33.9% and 23.2% in women in the general population. Higher rates of raised waist to hip ratio (WHR) and waist circumference were also found among African Caribbean and Sub-Saharan African women than among women in the general population. By contrast, African Caribbean men had similar rates while Sub-Saharan African men had lower rates of overweight and obesity than their White male counterparts. The prevalence of raised WHR and raised waist circumference were lower in both African Caribbean and Sub-Saharan African men than in their UK general population counterparts [97]. Higher rates of overweight have also been found among African Caribbean and Sub-Saharan African adolescents in the UK [101]. Similar higher rates have also been reported among African descent women in other European countries [98,100]. In the SUNSET study [98], 33.4% of the African Surinamese women were overweight and nearly 43% were obese compared with 40.2% overweight and 14.3% obesity in White-Dutch women. In their study comparing the Ghanaian population in the Netherlands with their counterparts in rural and urban Ghana, Agyemang and colleagues found Ghanaian migrants in the Netherlands to have an overly higher prevalence of overweight and obesity (men 69.1%, women 79.5%) than their urban (men 22.0%, women 50.0%) and rural (men 10.3%, women 19.0%) counterparts in Ghana [100]. Recent USA studies also show a higher prevalence of overweight and obesity among African American men and women compared with their White American counterparts in the USA [102]. Evidence also indicates that overweight and obesity are on the increase in Africa especially among women. A recent systematic review found that the prevalence of obesity in urban West Africa more than doubled (114%) over 15 years, with the increase accounted for almost entirely in women [103].

##### Causation

The possible reasons for the increased overweight and obesity among populations of African descent women are unclear. Obesity is, however, the result of an imbalance between energy intake and energy expenditure. Increases in the intake of fat and sugar as well as sedentary lifestyles have been linked to the rising epidemic of obesity [104,105]. In Luke et al's study, between 60% and 80% of the variance in adiposity between Nigerians and African Americans was explained by differences in activity energy expenditure or total daily energy expenditure [106]. In Harding and colleagues' work, excess overweight among African Caribbean and Sub-Saharan African girls in the UK was associated with adverse dietary behaviours [101]. Interestingly, current data seem to suggest that African Caribbean and Sub-Saharan African older women have favourable dietary behaviour, and do more physical activity than their White counterparts [97] despite their higher prevalence of obesity.

Cultural perceptions regarding overweight and obesity may also play a role in the increasing prevalence of overweight and obesity among these populations. In most African societies, being overweight or obese was and still is, at least in some part, associated with prestige, happiness and good healthy living, especially in women [107]. Many older people of African descent in Europe came at a time when these perceptions were very strong. It is possible that they have held on to these perceptions in Europe, which might be associated with a high rate of overnutrition and subsequently higher prevalence of obesity [100]. This, indeed, requires further studies.

##### Management

The increasing prevalence of overweight and obesity among populations of African descent especially in women underscores the urgent need to tackle this problem among these populations in Europe. Weight loss can improve or prevent many of the obesity-related risk factors for CVD. The optimal management of overweight and obesity begins with a combination of diet, exercise and behavioral modification. Addressing the obesity problem among populations of African descent may require culturally tailored approaches especially among the older generation. The perception of ideal body weight may differ between the older groups and their European-born children [108]. Hence obesity prevention initiatives need to be culturally tailored to prevent potential conflict of perceptions between the older and the younger groups. These approaches need to be validated and assessed to consider cultural acceptability, which is likely to affect uptake and compliance.

#### Physical activity

##### Burden

Physical inactivity represents an independent risk factor for CVD [109] and exercise is recommended to prevent CVD and promote and maintain healthy living [110,111]. The available data show important differences in physical activity levels among different ethnic groups. Evidence from the HSE 2004 shows that African Caribbean (31%) and Sub-Saharan African (29%) women were more likely than women in the general population (25%) to achieve the recommendations of participating in activity of moderate to vigorous intensity [97]. The rate in African Caribbean men (37%) and Sub-Saharan African men (35%) were similar to the men in the general population (37%). The Dutch data, [66] by contrast, suggest that African Surinamese were less likely than their White-Dutch counterparts to achieve the recommendations of participating in physical activity.

##### Causes

The high rate of physical activity levels reported among populations of African descent women in the UK contrasts the higher rates of inactivity related conditions such as obesity [97]. The reasons for this finding are unclear. It may be that because many African descent women are obese and have high rates of other risk factors such hypertension and diabetes; they may be more motivated than women in the general population to engage in physical activities. It may also well be that the heath education messages on physical activity are getting through to these communities in the UK. The lower rate of physical activity levels among African-Surinamese in the Netherlands may relate to several factors such as cultural differences in the representation of physical activity, the cost of engaging in physical activity, and insufficient skills to carry out the recommendations. In one Amsterdam study, several Ghanaians and African Surinamese hypertensive patients reported lacking sufficient skills and experience to carry out some of the physical activities (e.g., swimming and bicycle riding) recommended by their general practitioners (Beune E et al. unpublished data).

##### Management

A physically active lifestyle delivers significant physical and mental health benefit. Regular physical activity is recommended in the early school years and throughout life. However, the enablers and inhibitors of physical activity may differ between ethnic groups due to differences in social, cultural and individual factors. Strategies to improve physical activity among populations of African descent in Europe should be comprehensive and culturally tailored.

#### Dietary habits

##### Burden

Consumption of fruits and vegetables can protect against the development of CVD [112,113]. High fat intake raises cholesterol levels; and high cholesterol levels have been associated with obesity, abdominal obesity, and type II diabetes [114]. Dietary habits differ considerably among ethnic groups. The available evidence seems to suggest that fruit and vegetable intake is higher in populations of African descent than their European counterparts in Europe. In the HSE 2004 [97], African Caribbean men (32%) and women (31%) and Sub-Saharan African men (31%) and women (32%) were more likely than men (23%) and women (27%) in the general population to meet the recommended guidelines of consuming five or more portions of fruit and vegetables a day. High fat intake was also lower in the African groups than in the general population. The use of salt in cooking was conversely higher in African Caribbean and Sub-Saharan African men and women than in the general population. One study in the Netherlands also found that African Surinamese group scored higher on overall diet quality than the White-Dutch group [115]. Although African-Surinamese group scored higher on overall diet quality than their White-Dutch counterparts, fruit and vegetable intake were lower than recommended [115]. There are important differences between the older adults and the younger groups. In the HSE 2004, [97] the fruit intake among the younger African Caribbean age group (16–34 years) was similar to that of the general population. The older African Caribbean age group, by contrast, had higher fruit and vegetable intakes than their general population counterparts. Harding and colleagues also found that African Caribbeans and Sub-Saharan Africans boys and girls were more likely to skip breakfast and engage in other poor dietary practices than their White peers in the UK [116].

##### Causation

Following migration, many ethnic minority groups change their eating habits, combining parts of their traditional diet with some of the less healthy elements of the European diet [117]. Age and generation have been identified as the two major factors that determine the extent to which ethnic minority groups change their diets [117]. A number of studies in the UK, France and Spain have reported some departures from traditional African and African Caribbean diets following migration, especially among the younger generations [118,119]. In France, Caius and Benefice [118] found that the dietary habits of the West Indian adolescents were similar to their French peers. Others have, however, found no relationship between age or acculturation and dietary habits [115]. Other factors that may influence dietary habits include the proportion of income spent on food, availability of food, religion, and food beliefs [117].

##### Management

Changing diets of ethnic groups have resulted in major health concerns such as diabetes and obesity [117]. Post-migration dietary changes, especially among the younger age groups of African descent, together with the high rates of diet-related conditions present a huge challenge in reducing the risk of diet-related diseases among these populations. The available data seem to suggest, however, that the older people of African descent have favourable dietary habits despite their high rates of dietary related conditions such as obesity [97]. Although many people of African descent may report maintaining their traditional diet, it is possible that the preparation, serving practices, and eating habits have changed after migration [117]. Many dietary assessment questionnaires have also not been critically assessed for their suitability in these groups [97]. Hence the nutrient intakes of these groups need to be interpreted with caution. The mismatch between the self-reported dietary behaviour and the dietary related conditions clearly emphasise the need for further studies to critical examine changes in food preparation, serving practices and eating habits following migration among these populations in Europe.

#### Cigarette smoking

##### Burden

Tobacco smoking is an established risk factor for CVD through a variety of mechanisms [120]. There is an important heterogeneity between the populations of African descent in Europe. In the HSE 2004 [93] the rates of smoking among African Caribbean (men 25%, women 24%) were similar to that of the general population (men 24%, women 23%). Sub-Saharan African men (21%) and women (10%) had lower rates than that of the general population. The Dutch study [98] found a higher prevalence of smoking among African-Surinamese men (56%) than among White-Dutch men (44.9%). African Surinamese women (33.8%) were, however, less likely than White-Dutch women (44.3%) to smoke.

##### Causation

The explanations for the different patterns of smoking behaviour among populations of African descent are unclear. Differences in culture, socio-economic status and the level of acculturation may play a role. In most African societies, it is socially unacceptable for women to smoke and this may reflect the lower prevalence of smoking reported among Sub-Saharan African women in the UK. Socio-economic position in relation to smoking is inconsistent. In the HSE 1999 [27], the relationship of social class and equivalised household income to cigarette smoking was the same for women as for men in the general population. However, among African Caribbeans, there were no relationships between cigarette smoking and either social class or household income among women.

##### Management

Intensive behavioural interventions such as individual counselling, group counselling, telephone counselling and minimal clinical intervention (brief advice from a healthcare worker) can result in substantial increases in smoking cessation. Smoking cessation interventions may yield different results in smokers of ethnic minority groups. Effective strategies are needed to reduce tobacco use among populations of African descent in Europe, and thus diminish their burden of tobacco-related diseases and deaths [121]. Given the huge heterogeneity within the populations of African descent, preventive programmes may need to be culturally tailored to have an effect. Producing culturally sensitive information may help to raise awareness of the additional links between tobacco use and heart disease, oral cancers and respiratory disease [122].

#### Alcohol consumption

##### Burden

Epidemiological studies have suggested that heavy drinking constitutes a severe risk for CVD, but that low levels of consumption can have a protective effect against CHD mortality [123,124]. There has been little research on alcohol consumption among minority ethnic groups in Europe, and recent studies suggest that consumption levels tend to be lower among these groups than among White people. In the HSE 2004 [97], among both sexes, African Caribbean men and women (15% and 21% respectively) and Sub-Saharan Africans men and women (32% and 45%) were more likely than the general population men and women (8% and 14%) to be non-drinkers. African Caribbean and Sub-Saharan African men and women were less likely than the general population to report drinking on 3 or more days a week. African Caribbean and Sub-Saharan African men and women were less likely than their general population counterparts to exceed government recommendations on daily drinking amounts (i.e. 4 units for men and 3 units for women). They were also less likely than the general population to binge drink. Despite the comparatively low rate in the African decent groups, large proportions of African Caribbean men (20%) and Sub-Saharan African men (19%) drank enough to be classified as binge drinkers. Low prevalence of alcohol consumption has also been reported among African Surinamese men and women than among their White-Dutch counterparts in the Netherlands [98]. The contribution of alcohol consumption to ethnic differences in CVD is unclear. Studies indicate that low levels of consumption can have a protective effect against CVD [123,124]. It is possible that the relatively low prevalence of alcohol consumption among populations of African descent may contribute, at least in part, to the higher rate of CVD.

##### Causation

The explanations for the low prevalence of alcohol consumption among populations of African descent in Europe are unclear. Cultural and religious differences in the perception of alcohol use may play a role. For example, Islam prohibits alcohol use. Some African descent people in Europe are Muslims and therefore may not drink alcohol at all while others may drink but not report it for fear of stigmatisation [125].

##### Management

Treatment for alcohol dependence benefits both individual and society by improving individual health, productivity and quality of life. Although alcohol use is comparatively low in populations of African descent, large proportions of African Caribbean and Sub-Saharan African men still consume more than the government recommended daily drinking amounts and therefore need to be targeted for preventive care and advice on alcohol moderation [126].

#### Psychosocial stressors

##### Burden

Psychosocial stress is a risk factor for CVD morbidity and mortality [28]. Research on ethnic differences in psychosocial stress has produced largely conflicting results [27,127-130]. In Shaw et al's study, anxiety disorders were less common among African Caribbeans than among White people. Depressive disorders were, however, more common among African Caribbean women than White women [127]. Weich and colleagues found no differences in the prevalence of anxiety and depression between African Caribbeans and White people [128]. Wadsworth and colleagues' recent study, by contrast, showed that more African Caribbean respondents reported high work stress than either White counterpart [129]. In the HSE 1999, the proportion of African Caribbean women with high GHQ12 was significantly greater than women in the general population [27]. More recently, Maynard and colleagues assessed psychosocial well-being among youth of African and European descent in the UK, and found that African boys and girls reported the most favourable psychological wellbeing compared with their European counterparts [130]. Others have also found important differences within the African groups. In Maginn and colleagues' study, the rates of common psychological problems as assessed by GHQ were lower in Sub-Saharan African patients than in White patients, but there were no differences between African Caribbean and White patients in the UK [131].

##### Causes

The reasons for these inconsistent results are unclear although others have argued that the screening instruments in these studies may not be valid among these populations [132]. These inconsistent results are surprising given the higher rates of factors that may influence psychosocial stressors such as discrimination, high rate of unemployment, poverty, poor housing and lack of social support [27,97,125,133]. Evidence suggests that experience of racism is central to the lives of many ethnic minority people [125]. Findings from the UK Fourth National Survey of Ethnic Minorities showed widespread experiences of racial harassment and discrimination among ethnic minority people in the UK [125]. There was also a widespread belief that employers discriminated against ethnic minority job applicants [133]. The reported racial discrimination among African Caribbean female respondents was strongly associated with perceived work stress [129]. Similar findings have also been reported among African Americans in the USA. One study found that eighty percent of African American respondents in a USA study reported experiencing racial discrimination at some time in their lives [134]. Many of populations of African descent in Europe are also socially isolated. In HSE 1999, African Caribbean men and women were more likely than men and women in the general population to be classified as having a severe lack of social support [27].

##### Management

Recent findings indicate a significant level of unmet need for the treatment of psychosocial disorders and variable contact with general practitioners (GP) [135]. The increasing cultural and social diversity in Europe will affect need and access. Much attention has been paid to psychotic disorders among ethnic minority groups especially among African Caribbeans, but rather less attention has been paid to common mental health problems [131].

Evidence suggests that patients of African descent attend their GPs for consultations as frequently as White patients [2], but are much less likely to be referred for psychological therapies [136]. Evidence also suggests important differences within the African groups and emphasise the need to distinguish between these groups if progress is to be made on this topic. In Marginn et al's study [131], the detection and management of common mental disorders in African Caribbean patients were similar to that in White patients, but the Sub-Saharan African patients were less likely to be detected and to receive active management compared with White patients. The difference observed between the two African populations may reflect cultural differences between these groups. Sub-Saharan Africans with psychological problems may be less likely than their Caribbean counterparts to attend their GP, and may be less willing to speak to them about these problems [136]. This important difference within the African groups reiterates the need to recognise the important heterogeneity within populations of African descent in Europe [13].

### Emerging vascular risk factors

Traditional risk factors may not explain all cardiovascular events that occur. Epidemiological studies have explored a range of novel risk factors in an attempt to improve the prediction of future CVD. These novel factors include markers of inflammation and haemostasis. Of these factors lipoprotein(a) levels, total plasma homocysteine levels; fibrinolytic function as assessed by levels of tissue-type plasminogen activator and plasminogen activator inhibitor antigens; and inflammatory markers, such as fibrinogen and high sensitivity C-reactive protein (CRP) have received most attention [137]. Differences in biomarkers for inflammation and haemostasis might contribute to the observed ethnic differences in cardiovascular risk [138]. Data relating these novel factors to CVD risk principally come from White populations with limited information available across ethnic groups and particularly among populations of African descent in Europe. Some data from the UK suggest no apparent differences in CRP concentrations [139,140], but fibrinogen levels have been demonstrated to be lower in both West Africans and African Caribbeans than in their European counterparts in the UK [141]. A population-based study of multiethnic middle-aged men and women in the UK found slightly lower levels of circulating total homocysteine in both West Africans and African Caribbeans living in England compared to White people [142]. There is a clear need to include more people from ethnic minority backgrounds in future studies on these novel factors to improve on the current evidence available.

### Access and quality of care

Inequalities in access to and quality of care might also partly contribute to the observed high prevalence of stroke, hypertension and diabetes in populations of African descent in Europe. The UK data suggest that in general, a high proportion of people from most ethnic groups appear to be registered with a general practitioner – with registration rates of 99–100%, except for relatively low registration rate in African Caribbean men (96%) [143,144]. GP consultation rates have also been shown to be higher in all ethnic groups than in the UK general population except for Chinese [143,145-147]. The Dutch data also suggest higher GP consultation rates in all ethnic minority groups than in White-Dutch group [148,149]. Despite the higher GP consultation rates and free universal health care system at the point of access, a population-based study conducted to assess the quality of diabetes care and intermediate clinical outcomes observed that African Caribbean were significantly less likely to meet national treatment targets for diabetes, BP and total cholesterol compared to the White British group [90,150]. Information on differences in health care use regarding specialised procedures such as cardiac catheterization, coronary-artery bypass graft surgery, angioplasty and carotid endarterectomy is very limited in Europe. Evidence from the USA shows important differences in these procedures between African American and White Americans [151-153]. A study of residents in the ARIC study communities found African Americans hospitalised for definite MI to be two to three times less likely than White Americans to have received invasive cardiac procedures [151]. The differences in the rate of procedures received by African Americans and White Americans persisted even when the effects of disease prevalence, co-morbidities and other covariates had been taken into account. There is a clear need for further studies to determine whether ethnic inequalities in specialised invasive procedures exist, and if so, the extent to which these differences in these procedures contribute to CVD morbidity and mortality risk differentials. Current evidence suggests that ethnic minority groups are under-represented in clinical trials especially in Europe [154,155]. Ranganathan and Bhopal's systematic review shows a shortage of information from cardiovascular cohort studies on ethnic minority populations in Europe [155].

It is worth mentioning that the cause of inequalities in access to services may depend on a wide range of factors including knowledge of services and how to use them, health beliefs and attitudes, language barriers, the sensitivity of services to differing needs and the quality of care provided. These raise the key issues for health professionals of effective communication, awareness of attitudes, culture, stereotyping and racism within consultations and broader aspects of health service delivery for ethnic minority groups [156,157].

### Strengths and limitation

The main strength of our current paper is that it provides a comprehensive overview of the current understanding of the epidemiology of vascular disease and related risk factors among populations of African descent in Europe. There are also limitations. We carried out a comprehensive review of literature but did not systematically assess the quality of papers as would be done in a systematic review. Nonetheless, we had been fairly careful in ensuring that all major papers relevant to the issue have been cited. Many of the conclusions were also based on published systematic reviews and meta-analyses. Most of the studies came from only the UK and the Netherlands. Given the important heterogeneity within populations of African descent, there is an urgent need for studies among these populations living in other European countries. Data are also scant on some aspect of CVD and risk factor such as the emerging risk factors, management of some risk factors such as cholesterol and health care use regarding specialised procedures. In addition, the mortality data are largely based on pragmatic categories such as country of birth, which can be misleading [158]. Country of birth may reflect ethnicity reasonably well among some ethnic groups [17], but is likely to be an unreliable proxy measure of ethnicity for other groups. The Dutch Surinamese population, for example, is composed of multiple ethnicities (African descent, South Asian descent and others) [17]. As a result, the mortality data among African-Surinamese population in the Netherlands is not reliable. In addition, very little information was available on additional indicators of ethnicity, including migration history and length of stay in the residing country. This type of information is necessary to obtain more insight into the background of the patterns of ethnic differences, including issues such as the way these differences develop with increasing length of stay.

## Conclusion

This outline clearly indicates that hypertension and diabetes are higher in populations of African descent than in their European counterparts, which may contribute to the high rate of stroke among these populations in Europe. The relatively low rate of CHD may be explained by the low rates of other risk factors including a more favourable lipid profile and the low prevalence of smoking. The risk factors are changing, and on the whole, getting worse especially among African women. The UK African descent populations are significantly less likely to meet national treatment targets for diabetes and BP compared to the White British group. The BP control among African Surinamese in the Netherlands, especially men, is unacceptably low. The high rate of stroke will decline if high BP control improves among these populations in Europe. These findings clearly indicate that urgent action is needed in Europe to address these inequalities.

The effectiveness of CVD interventions in populations of African descent health needs to be established. This will require cohort studies and clinical trials to determine the relative contribution of vascular risk factors in these groups, and to help guide the prevention efforts. There is also an urgent need for trials to evaluate clinical outcomes among these populations in Europe. This is highly relevant because preventive strategies developed and tested in European populations may not apply to ethnic minority groups because of cultural differences, language barriers, poor education levels and poor social relations. Strategies may have to be specifically developed, validated and assessed to consider both cultural acceptability, which is likely to affect uptake and compliance, and underlying susceptibility, which may affect the effectiveness of preventive and therapeutic options in different ethnic groups. Lastly, more efforts are also needed to improve data quality by recognizing the important heterogeneity within African descent populations in Europe and between the first and the second generations.

## Competing interests

The authors declare that they have no competing interests.

## Authors' contributions

CA and JA drafted the manuscript with major contributions from ADA, RB and KS. All were involved in critical revision of the manuscript. All authors read and approved the final manuscript.
